# Clinical, Epidemiological, Diagnostic, and Therapeutic Patterns in Anti-NMDAR Catatonia: A Systematic Review of Case Reports

**DOI:** 10.1192/j.eurpsy.2026.11050

**Published:** 2026-07-31

**Authors:** I. V. Meira-Lima, R. N. Velloso

**Affiliations:** 1 Department of Clinical Medicine, Universidade Federal do Rio Grande do Norte (UFRN), Natal; 2 Department of Radiology, Universidade Estadual de Campinas (UNICAMP), Campinas, Brazil

## Abstract

**Introduction:**

Catatonia is a frequent and severe manifestation of anti-NMDAR encephalitis, often refractory to treatment. Its initial presentation may mimic psychiatric disorders such as schizophrenia or mania, delaying diagnosis and worsening prognosis. Identifying associated clinical and epidemiological patterns may improve diagnostic and therapeutic strategies, reducing sequelae and mortality.

**Objectives:**

To identify clinical and epidemiological patterns, as well as diagnostic and therapeutic approaches for catatonia associated with anti-NMDAR encephalitis reported in case studies, with emphasis on initial manifestations, ancillary tests, treatments, and outcomes.

**Methods:**

A systematic review of case reports was conducted in PubMed, Embase, and Google Scholar using the terms *“Catatonia”* and *“Anti-NMDA”* in title/abstract. Exclusion criteria were duplicates, reviews, commentaries, and abstracts without detailed clinical data. The final sample comprised 65 patients from 52 articles in multiple languages.

**Results:**

Of 65 patients, 78.5% were female, aged 2–78 years (mean 23.7). Adolescents (38.5%) and young adults (47.7%) predominated. Initial symptoms included acute psychosis (32.3%), insomnia (20.0%), seizures (13.8%), and bizarre behaviour (12.3%). Children more often presented neurological onset (seizures, confusion), while adolescents displayed psychiatric onset (psychosis, catatonia). Adults showed mixed patterns, and older patients had atypical features often misdiagnosed as neurodegeneration. Cerebrospinal fluid analysis contributed most to diagnosis (98%), followed by serum antibody panels (48%). EEG abnormalities were observed in 65%, and MRI was normal in 52%. Orofacial dyskinesia occurred in 34% and autonomic instability in 51%. Ovarian teratoma was found in 12%. First-line immunotherapy (methylprednisolone, IVIG, plasma exchange) was used in >80%, often requiring escalation to second-line agents (rituximab, cyclophosphamide). Electroconvulsive therapy (ECT) was applied in 26% with favourable outcomes. Most patients experienced gradual recovery, ranging from complete to partial, with no major gender differences.

**Conclusions:**

Anti-NMDAR catatonia shows a characteristic epidemiological and clinical profile with psychiatric and neurological prodromes. Early clinical suspicion and antibody testing are crucial for timely diagnosis. Treatment requires a dual approach: immunotherapy for the autoimmune process and benzodiazepines or ECT for catatonic symptoms. Prompt recognition and aggressive management improve outcomes. Greater awareness among psychiatrists is essential to avoid misdiagnosis and ensure timely interventions.

**Disclosure of Interest:**

None Declared

